# Enhanced Channel Calibration for the Image Sensor of the TuMag Instrument

**DOI:** 10.3390/s22062078

**Published:** 2022-03-08

**Authors:** Eduardo Magdaleno, Manuel Rodríguez Valido, David Hernández, María Balaguer, Basilio Ruiz Cobo, David Orozco Suárez, Daniel Álvarez García, Argelio Mauro González

**Affiliations:** 1 Department of Industrial Engineering, University of La Laguna, 38200 San Cristóbal de La Laguna, Spain; mrvalido@ull.edu.es (M.R.V.); alu0100070378@ull.edu.es (A.M.G.); 2 Institute of Astrophysics of Canary Islands, 38205 San Cristóbal de La Laguna, Spain; dhdez@iac.es (D.H.); brc@iac.es (B.R.C.); 3 Department of Astrophysics, University of La Laguna, 38200 San Cristóbal de La Laguna, Spain; 4 Institute of Astrophysics of Andalusia, 18008 Granada, Spain; balaguer@iaa.es (M.B.); orozco@iaa.es (D.O.S.); dalvarez@iaa.es (D.Á.G.)

**Keywords:** Sunrise mission, sensor calibration, FPGA, TuMag instrument, Low-Voltage Differential Signaling (LVDS) deserialization

## Abstract

The Sunrise missions consist of observing the magnetic field of the sun continuously for a few days from the stratosphere. In these missions, a balloon supporting a telescope and associated instrumentation, including a Tunable Magnetograph (TuMag), is lifted into the stratosphere. In the camera of this instrument, the image sensor sends its data to a Field Programmable Gate Array (FPGA) using eight transmission channels. These channels must be previously calibrated for a correct delivery of the image. For this mission, the FPGA has been exchanged for a newer and larger one, so the firmware has been adapted to the new device. In addition, the calibration algorithm has been parallelized as the main innovation of this work, taking advantage of the increase in logic resources of the new FPGA, in order to minimize the calibration time of the channels. The algorithm has been implemented specifically for this instrument without using the Input Serial Deserializer (ISERDES) Intellectual Property (IP), since this IP does not support the deserialization of the data sent by the image sensor to the FPGA.

## 1. Introduction

The Sunrise-3 mission pursues the uninterrupted observation of the sun’s magnetic field for a few days. With this objective, a telescope with its associated instrumentation will be carried to the stratosphere with the help of a balloon [1]. The National Program approved the Sunrise/IMaX project in 2002 as it was a strategic step towards the Polarimetric and Helioseismic Imager (PHI) for Solar Orbiter mission [2]. Sunrise’s first mission flew in 2009 and studied solar magnetism at minimal solar activity using the IMaX (Imaging Magnetograph eXperiment) instrument [3,4,5].

The second Sunrise mission took place in 2013. That year, there was a period of maximum solar activity, unlike in the first mission, in which there was a period of minimum solar activity [6]. The design of this mission did not have many changes: the telescope and the gondola were the same as in the first mission. The IMaX instrument had only a few minor changes and updates including the replacement of the FPGA (Field Programmable Gate Array). Only the filter system of one of the instruments was modified [7]. The scientific results of the Sunrise missions can be consulted at [6,7,8,9,10,11].

Now, Sunrise-3 is meant to observe the evolution of the magnetically coupled solar atmosphere at high spatial resolution rather than being a transient thought for a given phase of solar activity. A new, and enhanced version of the IMaX instrument has been designed, named TuMag (Tunable Magnetograph). The major conceptual change in the optics has been realized due to the tunable capability of the instrument filter. TuMag is capable of imaging the sun in several narrow bands. From these images, the information of the four polarization states can be extracted [12,13,14].

The design of the electronics is completely new in the TuMag instrument compared to the old IMaX [15]. The electronics update includes a miniaturization of the components, leaving more physical space for other instruments on board. In addition, electronic devices have more logical resources, allowing more complex tasks to be carried out [16,17,18,19,20].

The camera image sensor has been replaced compared to the previous instrument. The new image sensor is the GPIXEL GSENSE400-BSI [21]. The data acquired by the sensor are read using eight differential channels that supply 12-bit pixels at 300 Mbps. The management of the acquisition and reading and sending times of data are done through control signals that ships a 7-series Xilinx FPGA (Artix-7 device) [22]. All eight sensor channels must be pre-calibrated to obtain a correct data reading.

A Xilinx Spartan-6 FPGA is used in [23] to implement the image sensor driver including channel calibration [24]. The calibration is performed sequentially for each channel to save FPGA logic resources. In the TuMag camera, the control device uses a newer FPGA that supports the CoaxPress standard communication protocol for vision applications based on coaxial cables. Specifically, an Artix-7 FPGA containing a large number of logic resources has been used [25]. This allows channel calibration to be performed simultaneously, so the time taken to perform this task is greatly reduced. On the other hand, a component that introduces delays in the input signals must be used to perform the calibration. This component is substantially different from 6-series to 7-series Xilinx FPGA [26,27]. This implies that the calibration algorithm has been changed to adapt it to the new component with respect to the calibration method used so far in the literature [23,28,29,30], in which the algorithm detects two edges of the incoming signal to adjust the sampling time and, furthermore, the algorithm runs sequentially, one channel after another.

This work presents a new algorithm for calibrating the channels of the GSENSE image sensor as the main innovation of this work. FPGA devices have proven to be ideal for implementing algorithms for images coming from cameras for astrophysical applications [31,32,33,34,35,36,37,38,39]. We have had to design at a low level (structural level) because the data sent by the image sensor to the FPGA cannot be deserialized with the ISERDES IP available in the Vivado suite. That is why in this prototype, the algorithm has to be adapted to the Xilinx FPGAs of the 7-series, which presents new components in its architecture and a greater amount of logic resources compared to previous FPGAs. Taking this into account, improvements have been made at two levels: at a structural level, the algorithm has been modified to adjust the sampling time using a single edge of the incoming signal (instead two edges), adapting it to the new components of the Xilinx FPGA family 7. At the architectural level, the calibration algorithm trains all eight channels simultaneously rather than sequentially. This implies a significant decrease in the calibration time, at the cost of a greater use of FPGA hardware resources, but the new FPGA supports this increase as shown in the Results Section. This parameter could be important in environments and applications where channels need to be calibrated relatively frequently.

The rest of the present work is organized as follows: Section 2 briefly describes the TuMag camera, the prototype and the most relevant components. Section 3 details the firmware implemented in the FPGA to control the image sensor and send data to the frame grabber. Section 4 explains the three levels of the calibration task and the most relevant aspects of the new and enhanced channel calibration (using the new input delay component for Artix-7 and changing for a parallel calibration of channels). Then, in Section 5, we present the results. Finally, the conclusions are presented in Section 6.

## 2. The TuMag Camera

The camera of the TuMag instrument basically consists of a design of two PCBs, one of them contains the GSENSE400 image sensor and its power supply electronics and the other contains an Artix-7 FPGA in charge of controlling and receiving in the first instance the images acquired by the sensor.

**Figure 1:**
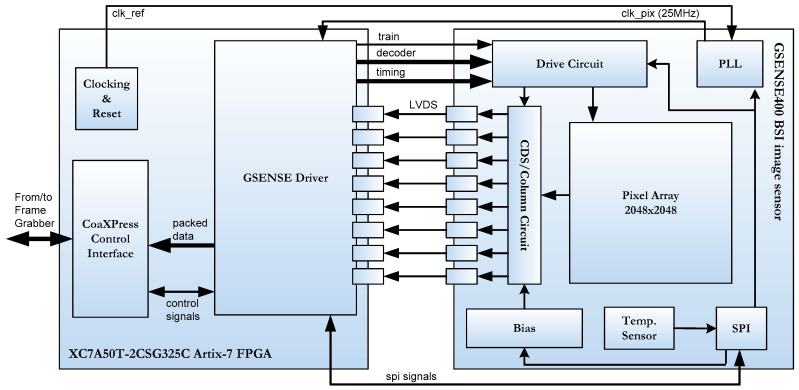
Diagram of the connection system between the image sensor and the FPGA device in the TuMag camera.

Figure 1 shows a schematic of the system consisting of the sensor and the FPGA, as well as their connections. As can be seen, the image sensor is configured by writing to a series of control registers via an SPI bus. Parameters, such as the training pattern, the sensor gain, the internal PLL multiplication and dividing factors, and so on, are selected in the register map accessed through this interface. In addition, the FPGA sends a reference clock signal with which the sensor sends a 25 MHz pixel clock that is synchronized with the image data that it sends in LVDS format to the FPGA. The *decoder* and *timing* signals in Figure 1 are used to determine both the reset and the reading of the different rows of the pixel array. The adjustment of the reset and reading time of each row by the FPGA driver determines the integration time, capturing the image sensor more or less light in order to form an image [21]. Finally, the *train* signal is used to calibrate each of the 8 differential channels with which the images are sent to the FPGA. As each pixel sent consists of 12 bits, data are sent at 300 Mbps. The training system is explained in Section 4.

**Figure 2:**
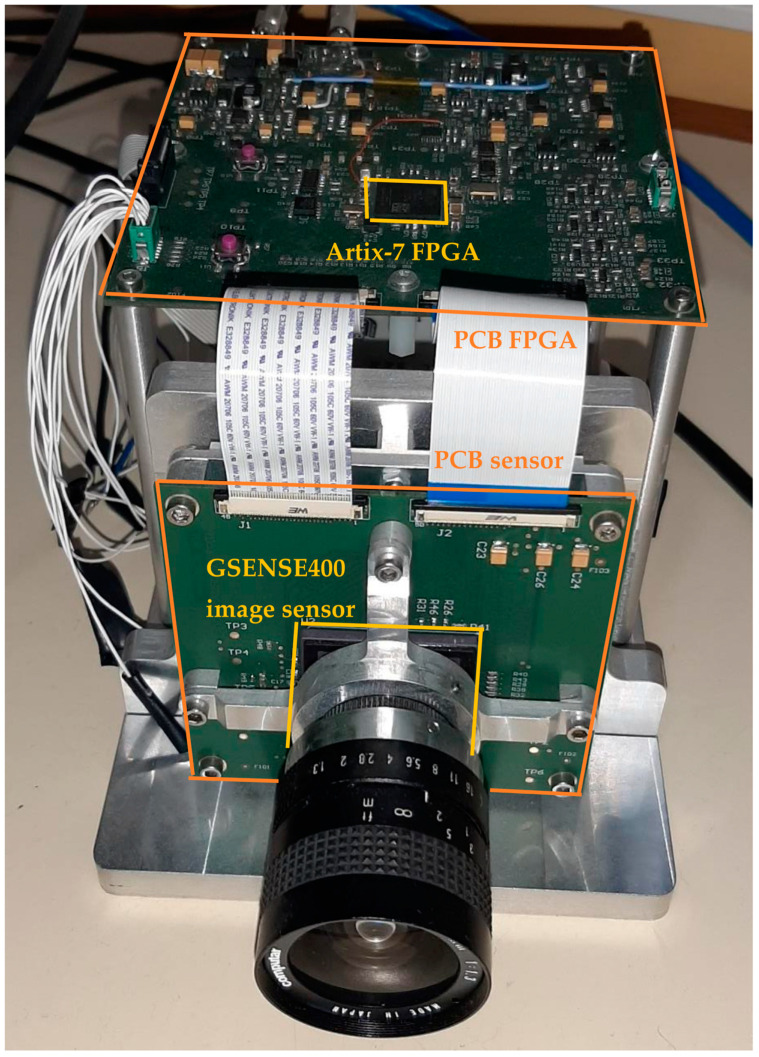
Picture of the first prototype of the TuMag instrument camera.

The prototype of the TuMag camera with which the development and debugging of the firmware was carried out consists of 2 PCBs (Printed Circuit Boards) as shown in Figure 2. The image sensor has been separated from the FPGA that communicates with this device. Thus, the vertically placed PCB includes the GPIXEL GSENSE400-BSI CMOS image sensor and the horizontally placed PCB includes an Artix-7 XC7A50T-2CSG325C FPGA [21]. The FPGA includes a CoaXPress communication IP in order to send the image data to the Data Processing Unit [22,40].

Basically, the image sensor has a resolution of 4 megapixel (2048 × 2048), 8 LVDS channels to send captured images and an SPI interface to configure the sensor operation. Each pixel has 12 bits and each pixel is sent at 25 MHz. Thus, the transmission speed is 300 Mbps through the 8 differential channels. These communication channels have to be calibrated before the image sensor operates in order for the data to be transmitted correctly. The calibration algorithm is implemented in the FPGA and includes controlled delays, bit rotations, and variable registers as shown below. In the standard mode operation, the image sensor works at 48 fps.

Artix-7 FPGA device consists of an array of Configurable Logic Blocks (CLBs) which are composed of two slices. These elements are connected to other similar blocks via programmable interconnects and switch matrices. Inside a slice, there are eight flip-flops and four Lookup Tables (6-LUT). Double data rate (DDR) is supported by all inputs and outputs. Any input and some outputs can be individually delayed by up to 32 increments of 78 ps, 52 ps, or 39 ps each. Such delays are implemented as IDELAY and ODELAY modules [22,26]. The number of delay steps has to be set by the calibration algorithm in order to receive the image data incoming from the sensor. These primitives make the design of serializer and deserializer circuits very straightforward and allows higher operation at speeds from 415 Mbps to 1200 Mbps per line [41].

The Artix-7 XC7A50T-2CSG325C FPGA has 32,600 6-LUT and 65,200 slice registers. The FPGA implementation makes the designed algorithm, flexible, customizable, reconfigurable or reprogrammable with advantages of well-customized, integration, accessibility and expandability. The system can be resized according to its needs taking advantages of the VHDL and verilog configurability. This device also has 150 input/output blocks (IOBs). Each IOB is configurable and can comply with a large number of I/O standards [24]. In this case, eight IOBs have been configured as LVDS inputs using DDR data reception with per bit-deskew.

## 3. TuMag Camera Firmware Overview

**Figure 3:**
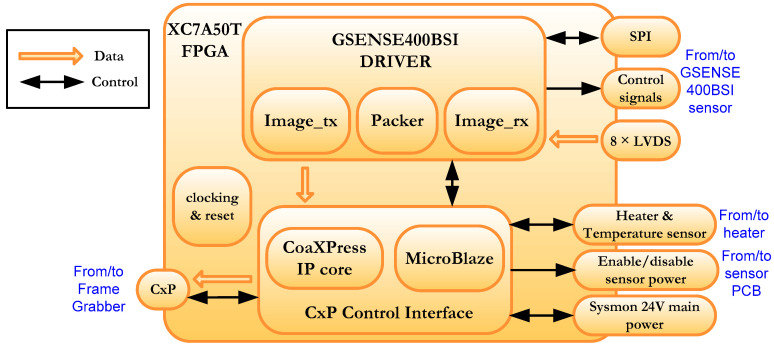
Camera FW device architecture.

Two main blocks can be distinguished in Figure 1, namely, the GSENSE400 driver and the CoaXPress (CxP) control Interface. A detail of the implemented firmware in FPGA is depicted in Figure 3.

The CxP Control Interface includes the Eurasys CoaXPress IP which allows communication with the Data Processing Unit using a coaxial cable [42]. The management of this IP is carried out using the embedded MicroBlaze soft-processor, which also monitors the main power and manages the sensor power and a heater [43,44,45]. Through this interface the data are sent at 3.125 Gbps.

The GSENSE400-BSI block is the other block of this system, responsible for the direct communication with the GESENSE400-BSI sensor. It manages several tasks such as:

1. Configuration of the sensor, through the SPI interface;
2. Generation of the control signals necessary to grab images from the sensor, such as the 19 control signals and the row address signal (decoder and timing signals in Figure 1);
3. Implementation of the image receiving interface (rx), based on 8 LVDS serial data channels from the camera that are converted to 8–12-bit parallel channels;
4. Channels calibration task;
5. Generation of the signals to arrange the received image;
6. Image ordering and packing;
7. Implementation of the image transmitting interface (tx) to CxP Control Interface.

**Figure 4:**
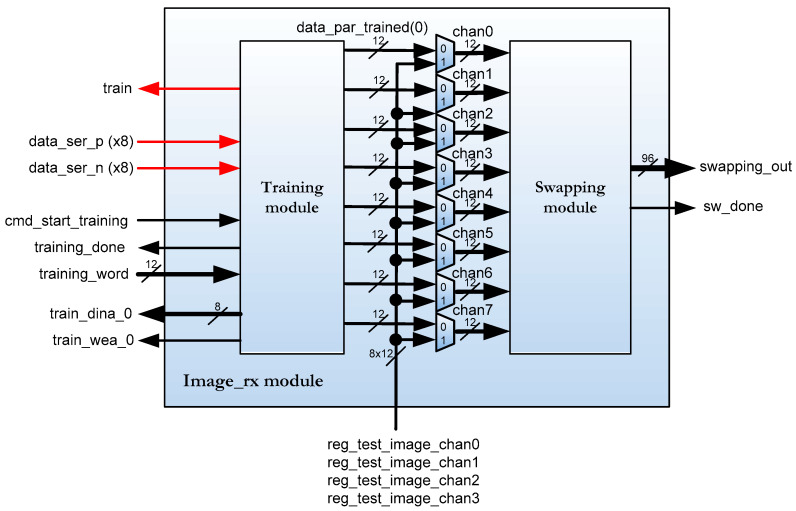
*Image_rx* block diagram.

*Image_rx* sub-module in Figure 3 implements the training algorithm to calibrate communication channels. Figure 4 depicts the *image_rx* sub-module. Red arrows are signals from/to the GPIXEL sensor.

The reception driver module of the sensor consists of two main modules. The first of these is the module called training, which receives data from the 8 differential channels. The second of them is the module called swapping, which performs the treatment of the channels to form an ordered image. The image sensor does not supply the image data in a row-by-column format. The GPIXEL sensor supplies image data in eight-channel format. This task is implemented in the swapping module [39]. Instead of entering the data image incoming from the sensor, it is possible to enter a test image to debug the design using multiplexers without using the image sensor.

**Figure 5:**
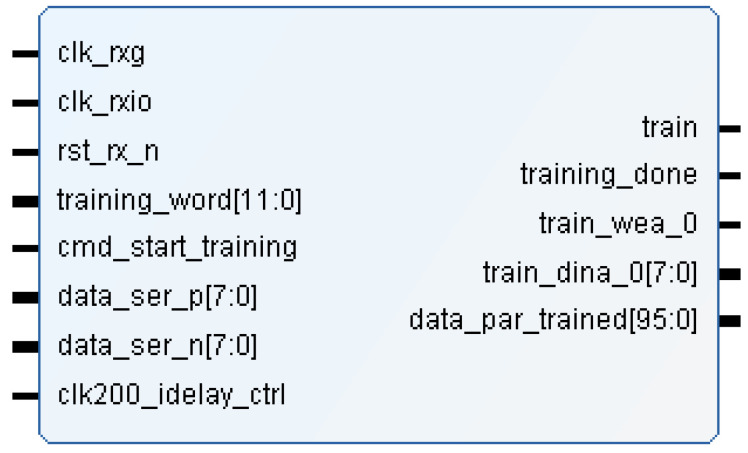
Module interface of training.v.

The training module is described in verilog code and it has the interface shown in Figure 5.

This sub-module calibrates the data channels in the FPGA so that the data are received correctly aligned (*data_ser_p/n* input ports). Each of the 8 channels includes a 12-bit deserializer with an adjustable input delay, called IDELAY2. Since data are transmitted at 300 Mbps, a 150 MHz clock is used to sample the incoming signal on rising and falling edges (*clk_rxio clock* signal). For this task, the IDDR (input dual data rate) components of the FPGA input blocks are used. These components are controlled by a component of the Artix-7 FPGA, called ICONTROL, which operates at 200 MHz (*clk200_idelay_ctrl* clock signal in Figure 5). These three components are new to the Xilinx FPGA 7 series [25,26]. Basically, a pulse on the *cmd_start_training* port indicates that channel calibration should be performed using the *training_word* value and the *train* port is set to one. When the calibration is finished, the calibrated and parallelized data are available on the *data_par_trained* port and the *training_done port* is set to one. Finally, *train_dina_0* and *train_wea_0* ports are used to send the data information for the calibration results.

## 4. Channel Calibration

**Figure 6:**
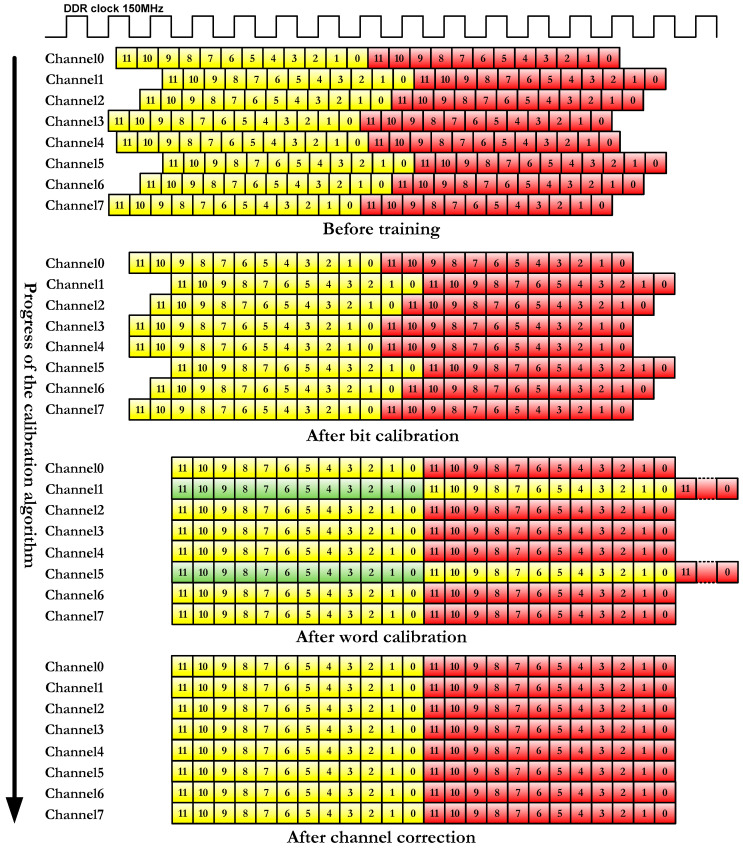
Calibration process.

The calibration of each channel is done in three steps as shown in Figure 6. The first step is the calibration of the bit, in which controlled delays are induced to ensure that the data are sampled in the center of the eye and not on one edge [41]. The correct reading of the bits is guaranteed with the previous step, but when inferring delays, it is possible that the 12 bits of each pixel are not aligned. Thus, in the second stage, the received data are rotated until they coincide with the control data previously agreed with the image sensor (*training_word* port in Figure 1). The third and last step is used to synchronize the channels so that all data are received in the same number of clock cycles. The IP RAM-based Shift Register is used to make this setting. All the parameters obtained in these three stages are written in a FIFO to later send them to the DPU to check the result of the calibration (*train_wea_0* and *train_dina_0* signals in Figure 4 and Figure 5). If any channel has not been calibrated correctly, the process is repeated.

Prior to performing this process, a training word is configured in the image sensor (for example, 0 × 98e). When the training module sends a train command to the image sensor (*train* signal set to one), it sends the training word on all channels all the time as pixel data. This constant pixel value is used to calibrate the channels according to Figure 6.

**Figure 7:**
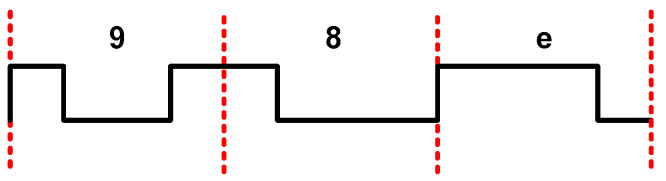
Data outcoming from image sensor to FPGA when train signal is set to high and training word is X“98e”.

As previously mentioned, the image sensor is configured to send a training word continuously on all channels when the *train* signal in Figure 1 is set to one. Figure 7 depicts data sent by the GSENSE400 using 12-bit X“98e” as training word.

For each channel, the first thing that is performed is the bit calibration in order to sample each received bit in the middle (Figure 6). This exact sampling point is achieved by delaying the input signal by a few picoseconds. When the received data change with respect to previous data, it means that an edge has been detected in the incoming signal.

**Figure 8:**
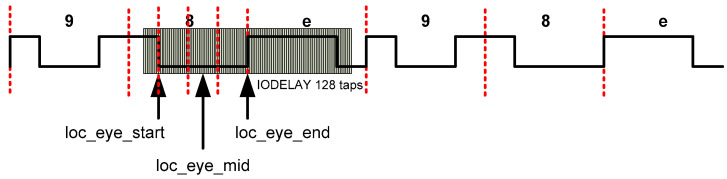
Bit calibration using IODELAYE2 component.

The component that performs these delays in the Spartan-6 FPGA is IODELAY2 [46]. Each IOB in the Spartan-6 FPGA contains this delay line that can be configured either for use as an input delay or output delay. This component can deserialize signals up to 1050 Mbps [47]. An 8-bit delay value allows delays from 0 to 255 taps to be achieved. The delay taps have an average value of 40 ps [23]. Two edges of the incoming signal are detected by delaying it using the IODELAYE2 component. The number of taps for each edge are *loc_eye_start* and *loc_eye_end* in Figure 8. With these tap values, the *loc_eye_mid* value is obtained.

Setting the delay line to *loc_eye_mid* delays the input data by exactly one half an input clock cycle, allowing data sampling in the middle of the input data eye. When the bit calibration is finished, the received word may not be aligned. The training word 98E has been chosen because the rotations of the bits produce different values: 98e, 31d, 63a, c74, 8e9, 1d3, 3a6, 74c, e98, d31, a63, 4c7. In word calibration, the received word is rotated until it matches the expected word. This number of rotations is the *loc_word* parameter. In the worst case, 12 rotations should be performed. Finally, a variable shift register adjusts the number of cycles for each channel so that all data are synchronized (*loc_chan* parameter).

At the end of each channel calibration, the calibration parameters of this iteration (*loc_eye_start*, *loc_eye_mid*, *loc_eye_end*, *loc_word*, *loc_chan* and *loc_nok*/*loc_ok*) are written into a memory. The software must read these data to ensure the success of the calibration or the need of another calibration. When the 8 channels are calibrated, the main FSM is set to the high *training_done* signal and goes to an idle state waiting for a new command for start training.

### 4.1. Migration of the Calibration Algorithm to Artix-7 FPGA

**Figure 9:**
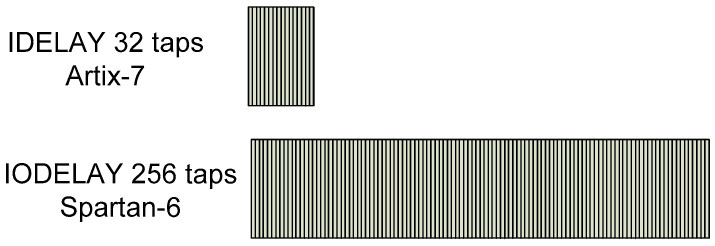
Comparison between IODELAY Spartan-6 FPGA and IDELAY 7-series FPGA.

**Figure 10:**
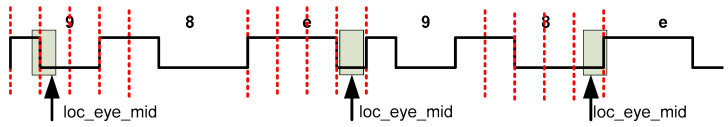
Edge search with the IDELAYE2 component.

The Xilinx FPGA family 7 represented a substantial change in the architecture and internal components of these devices compared to the previous ones. Because of this, upgrades of firmware implementations to newer FPGAs are not easy [48]. Several modifications were carried out with respect to the IMaX firmware for Spartan-6:

- Migration from Spartan-6 components to Artix-7 ad hoc components: substitution of IODELAY by IDELAY2, substitution of IDDR2 by IDDR. The 7-series devices have dedicated registers in the ILOGIC blocks to implement input double-data-rate (DDR) registers. This component is similar to the IDDR2 component of the Spartan-6 FPGA and direct replacement is possible [46,48]. However, the IDELAYE2 component of the Artix-7 FPGA differs greatly from its equivalent component of the Spartan-6 and direct replacement is not possible. The 6-series IODELAY component has a 256 tap-delay but the 7-series IDELAY2 has only 32 tap-delay [49]. Figure 9 depicts a comparison between both components;
- This time, the data capture mechanism is dependent on the IODELAY2 component instead IDELAY. The 6-series IODELAY component has a 256 tap-delay but the 7-series IDELAY2 has only a 32 tap-delay of nominally 78 ps and the minimum capture frequency = 78 × 31 = 2418 ps = 415 Mb/s. The frequency of the design is lower (300 Mb/s) and it could result in no edges at all being found. So, the previous algorithm using Spartan-6 FPGA has been modified to consider this situation;
- The bit calibration for Spartan-6 searches 2 edges but the modification for Artix-7 FPGA searches only one edge. If an edge is found in the delay line, then the final delay is statically set to be this value, ±16 taps. If no edge is found, the delay line is set to be 16 taps. In either case, the delay is set to be at least 16 taps away from the edge of the eye, which is acceptable at these lower bit rates [41]. Figure 10 depicts the 3 possible situations in the edge detection mechanism. At left, the edge is detected with taps less than 16 (for example 3), so 16 is added (set to sample a number of taps equal to 19). In the center, no edge is detected, so sampling is set at 16 taps. On the right, the edge is detected with a tap greater than 16 (for example, 20), so 16 is subtracted (sampling with 4 taps).

Regarding the calibration of the channels, this is still sequential, with state machines similar to the Spartan-6 FPGA implementation controlling the calibration. The most relevant change is made in the bit calibration process, where an edge is searched instead of two, and in the calculation of the number of sampling taps (*loc_eye_mid*) because the *loc_eye_end* parameter is not used (taps value for the second edge is also eliminated). It is important to note that the calibration time is reduced since the calibration being equally sequential, now the number of taps to be covered is reduced from 256 to 32. The value of the reduction depends on the detection of the edge, but it will always be less than the Spartan-6 based solution looking for 2 edges.

### 4.2. FPGA Concurrent Channel Calibration

The migration of the channel calibration supposes an acceleration of the algorithm caused by the change in the calculation method of the sampling instant in the bit calibration (see Figure 6).

The acceleration of the channel calibration algorithm is much more relevant if the channels are calibrated simultaneously rather than sequentially. This is achieved by using more logic resources of the FPGA. The motivation for the change of the algorithm at the cost of logical resources is due to the fact that the Artix-7 FPGA is larger than the Spartan-6 and an increase in the logic resources used for this task does not compromise the rest of the tasks that the firmware must perform (communication, control, configuration, and so on).

The training module has been separated into 2 hierarchical levels in order to carry out the parallelization. At the lowest hierarchical level, the deserializer of each channel is implemented with its own local training control. This sub-module is named *training_one_channel*. At the top hierarchical level, each channel is monitored and all calibration data are written after all 8 channels have completed the process.

**Figure 11:**
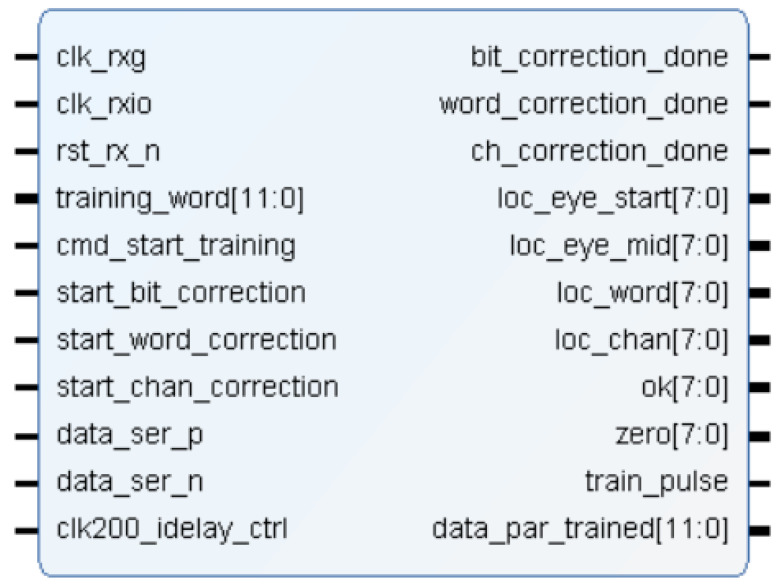
Module interface of training_one_channel.v.

The top level of the training module for concurrent calibration has the same block diagram and port descriptions that the sequential one (see Figure 5). The *training_one_channel* sub-module is described in verilog code and it has the interface shown in Figure 11. Table 1 also shows a brief description of each port of the module.

**Figure 12:**
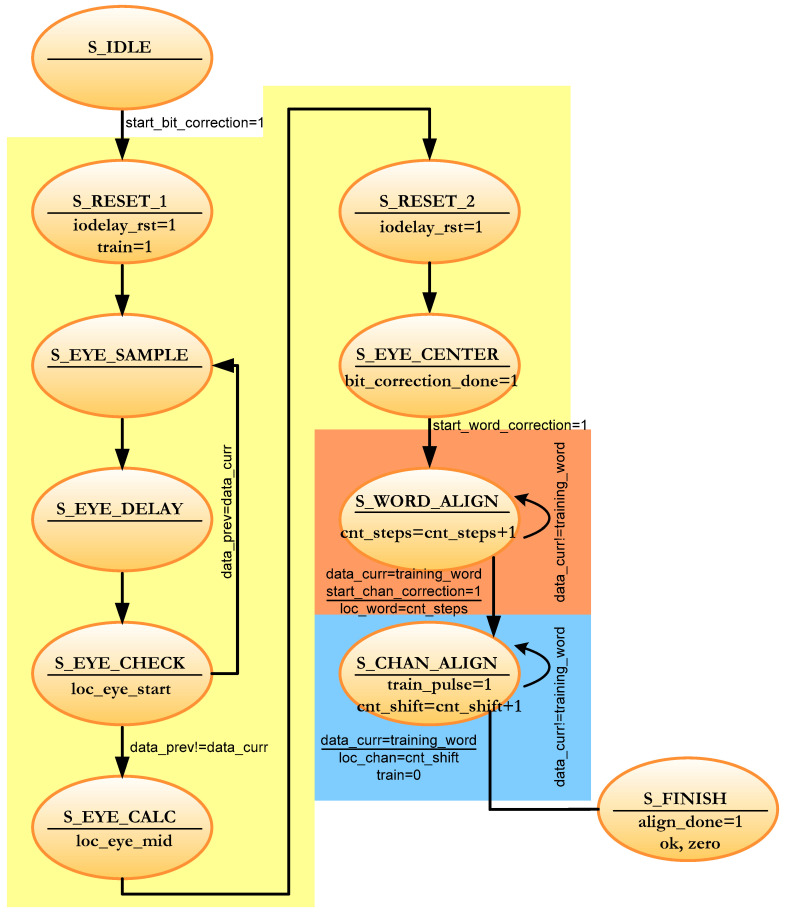
Simplified FSM for one channel calibration in the concurrent architecture.

Figure 12 depicts a simplified diagram of the local FSM of each channel for concurrent calibration. The yellow region corresponds to the bit calibration. The red region (S_WORD_ALIGN state) corresponds to the word calibration. The blue region (S_CHAN_ALIGN state) corresponds to the channel calibration. Now, the FSM must wait for the *start_bit_correction*, *start_word_correction* and *start_chan_correction* commands to start each of the three calibration levels. At the end of each calibration stage, a signal is sent to the general calibration control (in the upper hierarchical module). These signals are *bit_correction_done*, *word_correction_done*, and *chan_correction_done* in Figure 11. Note that the writing of the calibration results is not done in this sub-module. This task is carried out in the upper module as opposed to sequential calibration.

**Figure 13:**
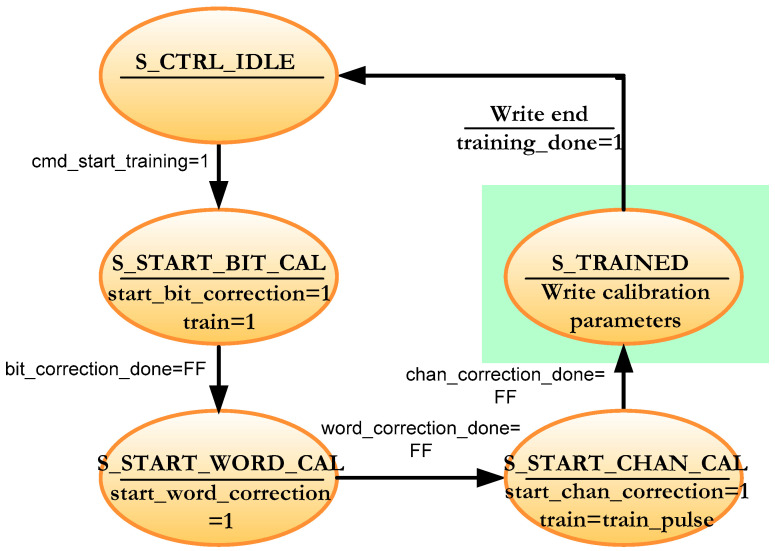
Simplified FSM for the overall calibration system in the concurrent architecture.

Figure 13 depicts the simplified state machine that controls the concurrent calibration of the 8 channels. The machine starts in the S_CTRL_IDLE state. When the *cmd_start_training* signal goes to high, the next state is S_START_BIT_CAL, which sets the *train* signal to 1, so it instructs the sensor to send the calibration word on all channels all the time. At the same time, the *start_bit_correction* signal is set to high, instructing the 8 sub-modules to begin the bit calibration in Figure 6. It remains in this state until all submodules indicate that they have finished the bit calibration (*bit_correction_done* = FF). When this happens, the *start_word_correction* signal is set to high in the S_START_WORD_CAL state, so the word calibration begins. As in the previous state, the machine remains in this state until all the sub-modules complete this calibration (*word_correction_done* = FF). Channel calibration begins in the S_START_CHAN_CAL state by setting the *start_chan_correction* signal to high and sending a pulse in the *train* signal. Each channel counts the number of clock cycles it takes to receive the training word to set the variable shift register with the correct depth. All channels report that they have finished the channel calibration (*chan_correction_done* = FF) and all the calibration parameters obtained are written into a FIFO. The green shaded area is equivalent to writing the calibration parameters for each channel in the sequential calibration but for all channels.

**Figure 14:**
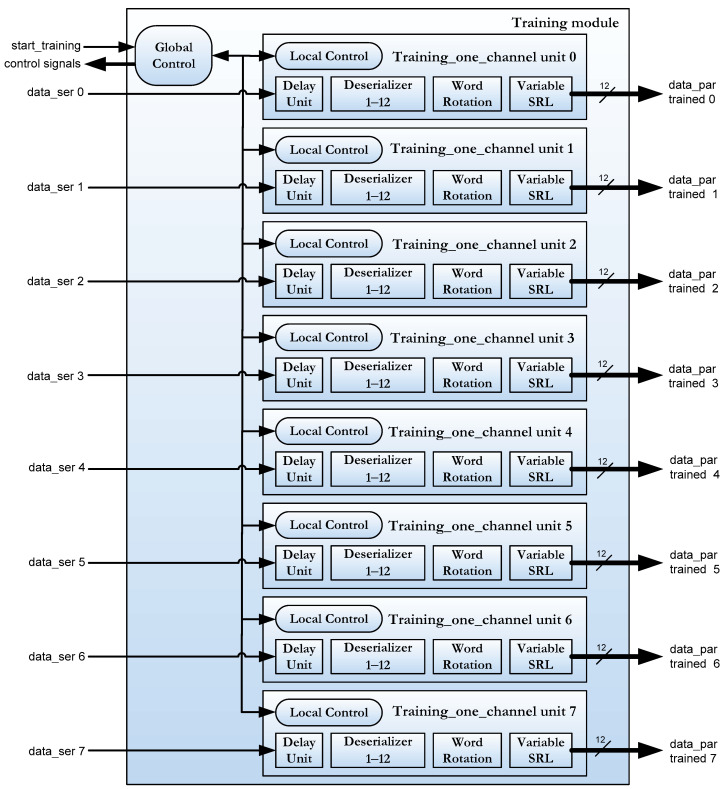
Block diagram of the concurrent channel calibration for the training module.

The data reception module in the FPGA consists of 2 sub-modules as mentioned in Section 3: the training module and the swapping module (see Figure 4). In the concurrent implementation of channel calibration, the training module has the architecture shown in Figure 14. Each channel now has its own local control logic module that runs the algorithm in Figure 12 and controls the calibration process for each channel. A global control module that manages the entire training module according to the algorithm shown in Figure 13 supervises these local logic sub-modules. The ports described in Figure 5 and Figure 6 are grouped in *the control signal* bus in the diagram of Figure 14.

## 5. Results

An enhanced channel calibration for the GSENSE400 image sensor of the TuMag instrument has been implemented. The implementation consisted of two steps: first, there was the migration of the calibration algorithm from the Spartan-6 FPGA to the Artix-7 FPGA, preserving the architecture of the module and adapting the design to the components of the 7-series of Xilinx FPGAs; and finally, the calibration algorithm was parallelized to minimize training time. The codes have been developed using Vivado 2017.4 for simulation, debugging and implementation.

As mentioned above, the migration of the calibration algorithm to the new FPGA resulted in a reduction in training time. This is due to the fact that the component that infers delays on the incoming signals, the IDELAY component, has a lower number of taps than the equivalent component of the previous FPGA. In addition, the algorithm had to be adapted to look for one edge in the incoming signal instead of two, as mentioned in Section 4.1.

Once the migration to the new FPGA had been carried out, the possibility of modifying the architecture of the training module was studied to calibrate the 8 channels of the image sensor simultaneously.

**Figure 15:**
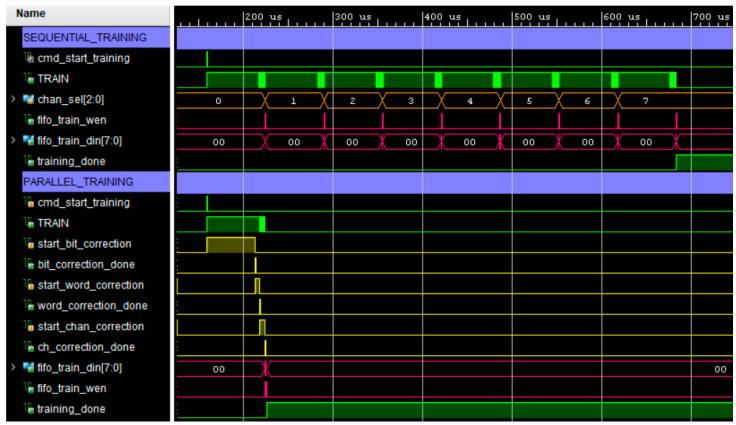
Comparison between the sequential and the parallel algorithm.

Figure 15 shows a comparison between the sequential algorithm and the parallel algorithm. The sequential algorithm calibrates the channels one after another using an 8-to-1 multiplexer. The *chan_sel* signal (in orange in the figure) selects the currently active channel. The calibration parameters are written at the end of the calibration of each channel (*fifo_train_wen* and *fifo_train_din* signals in magenta in the figure). The parallel calibration algorithm can be seen below the sequential algorithm in Figure 15. The operation of the parallel algorithm has been described in Section 4.2. All channels are in the bit calibration phase when the *start_bit_correction* signal is set to high. When all channels have finished this phase (*bit_correction_done* signal set to high), word calibration proceeds (*start_word_correction* signal is set to high) and ends when all channels set the *word_correction_done* signal to high. In the same way, the channel calibration of all channels starts simultaneously when the *start_chan_correction* signal is set to high and when all channels finish this stage, the *ch_correction_done* signal is set to high. At this moment, the calibration parameters of all the channels are written and the algorithm ends with the *training_done* signal set to high.

Table 2 shows the computed times measured for channel calibration using the three available architectures. There is a relevant reduction in the calibration time of the migration from the Spartan-6 FPGA to the Artix-7 FPGA. It is due to the component that infers the delay has 8 times fewer taps. In addition, this fact implies that the algorithm should look for an edge in the incoming signal instead of two edges, as mentioned in Section 4.1. In addition, for the same FPGA, the speed-up of the parallel implementation compared to the sequential one is 8.68, as expected.

The FPGA resources used by each of the three sub-modules are detailed in Table 3. It can be seen that the parallel implementation uses more resources than the sequential one. Parallel channel calibration uses 4.4 times more LUTs and 2.2 times more flip-flops. Even so, the amount of resources used is relatively low (less than 5% and 2%, respectively), so it is acceptable to speed up the calibration at the cost of this increase in logical resources. The remaining space on the FPGA is more than enough for the implementation of all the other control tasks that the FPGA must perform.

GSENSE400 is a relatively new image sensor with high dynamic range, high sensitivity and low noise. The few developments that they have currently been implemented for this sensor use FPGAs older than the Artix-7, such as the Virtex-4, Virtex-5 and the Spartan-6 [27,28,29,30]. These designs employ sequential calibration and have used an old development environment that Xilinx no longer supports (Xilinx ISE). Our implementation using an Artix-7 FPGA has been developed using Vivado, the current Xilinx tool. This makes it easy to upgrade the FPGA firmware with new IP libraries. Currently, there is an IP in Vivado for Xilinx 7-family FPGAs that automatically calibrates channels in a way that is easy for firmware developers. This IP is called ISERDES and is a deserializer of incoming data that includes calibration [50,51]. This IP only supports deserializing data of 1:2, 1:4, 1:6, 1:8, 1:10 and 1:14 using two ISERDES modules in a master–slave system, so it is incompatible with deserializer 1:12 that the driver needs to receive the incoming data from the GSENSE400 image sensor. Deserializers based on Artix-7 FPGAs can be found for other image sensors [52,53]. They use the ISERDES IP because they require 1:8 deserializers in their developments. This work results from the development of a very specific instrument. It is because of this that it is compared to previous versions of itself. The works that are referenced in the literature do not specify the calibration time or the FPGA resources that the deserializer uses together with the calibration of the channels. The comparison is difficult for developments using the ISERDES module, because the channel deserializers are not 1:12. A qualitative comparison of the design can be made according to [52]. Table 4 shows the comparison with a design that uses an Artix-7 to perform 1:8 deserialization using the ISERDES IP. In our design, the bandwidth per lane is limited by the GSENSE400 image sensor.

**Figure 16:**
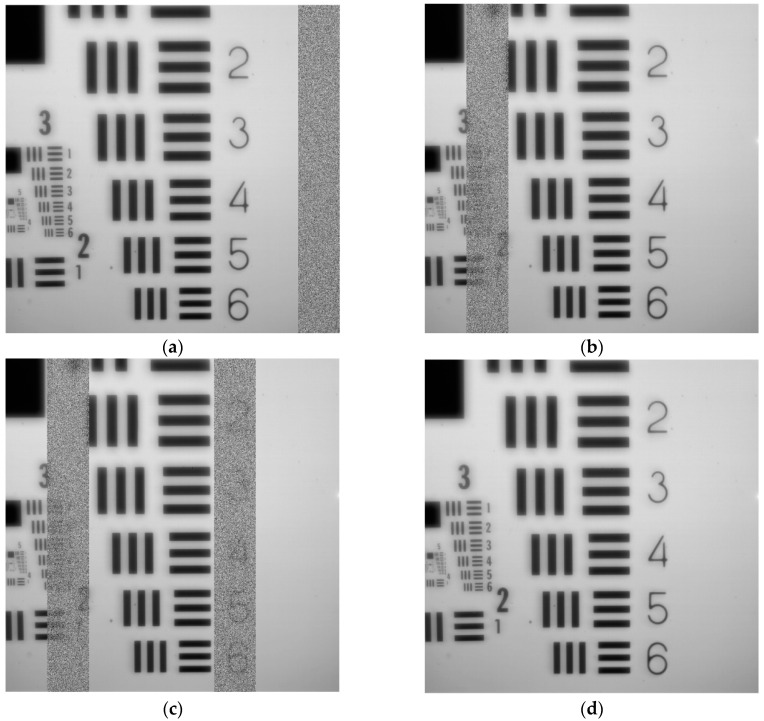
**A** 12-bit 2048 × 2048 image from GSENSE400 using the USAF pattern: (**a**) calibration error in channel seven; (**b**) calibration error in channel one; (**c**) calibration error in channels one and five; (**d**) successful calibration.

Figure 16 shows the results of acquiring an image with the prototype in which the USAF (United States Air Force) resolution test chart has been used. Figure 16a–c shows failed calibration workouts on channels seven, one; and one and five, respectively. A failed calibration is notified through the control signals detailed in Section 4. The calibration is repeated as many times as necessary until it is successful, as in the case of Figure 16d.

## 6. Conclusions

The concurrent calibration for the channels of the GSENSE400 image sensor has been successfully designed. The calibration process includes bit, word and channel calibration for each channel of the image sensor. Parallel calibration is 8 times faster than expected, as the sensor consists of 8 channels that are now calibrated simultaneously. Although the amounts of logical resources of the FPGA are greater than in the sequential implementation, these are not relevant with respect to the amounts of resources available in the Artix-7; so, there are no problems in parallelizing the training algorithm.

In general, it is preferable to use functional HDLs (hardware description languages) using FPGA-based design technologies. This makes the design independent of the technology (type of FPGA). However, the calibration algorithm has been carried out using specific components of the 7-series family Xilinx FPGAs, since the ISERDES IP cannot be used, so the algorithm has been designed using structural HDL. That is why the implemented algorithm is limited to Spartan-7, Artix-7, Kintex-7 and Virtex-7 FPGAs, being incompatible with FPGAs from other manufacturers and other FPGA families from Xilinx itself.

Technological advancements lead to the expectation that the next generation of image sensors will be greater than the current ones. An increase in the number of channels affects the design very little, as it is a modular and versatile design. The design is easily extrapolated to a greater number of channels to be calibrated.

## Figures and Tables

**Table 1:** Port description of training_one_channel.v module.

Port	Description
clk_rxg	25 MHz clock
clk_rxio	150 MHz clock for sampling channels
rst_rx_n	low level reset
training_word	word used for calibration task by comparison
cmd_start_training	command for start training task
start_bit_correction	command to start bit correction
start_word_correction	command to start word correction
start_chan_correction	command to start channel correction
data_ser_p/n	8-LVDS channels from the sensor
clk200_idelay_ctrl	200 MHz reference clock
data_par_trained	parallelized and calibrated data (8 channels)
bit_correction_done	flag for the end of bit correction
word_correction_done	flag for the end of word correction
ch_correction_done	flag for the end of channel correction
loc_eye_start	Tap value for the edge detection
loc_eye_mid	Tap value for sampling
loc_word	Number of rotations
loc_chan	Number of shift register
ok	Calibration channel ok
zero	Zero value (for debug)
train_pulse	Train pulse order for channel calibration
data_par_trained	parallelized and calibrated data (1 channel)

**Table 2:** Calibration times for the GSENSE400 image sensor.

Training Module	Time
Sequential calibration (Spartan-6)	4122.58 μs
Adapted sequential calibration (Artix-7)	524.88 μs
Concurrent calibration (Artix-7)	60.44 μs

**Table 3:** XC7A50T Artix-7 FPGA logic resources (available and used).

	Available Resources	Sequential Calibration	Concurrent Calibration
LUT	32,600	303 (0.93%)	1332 (4.09%)
Flip-flops	65,200	463 (0.71%)	973 (1.49%)

**Table 4:** Comparison of systems using the Artix-7 FPGA deserializer.

	Liu et al. [52]	Our System
System resolution	3840 × 2160	2048 × 2048
Maximum bandwidth per line	891 Mbps	300 Mbps
Deserialization ratio	1:8	1:12
Calibration implementation	ISERDES IP module	Structural HDL code
Hardware cost	Small	Small

## Data Availability

Not applicable.
